# Self-Reported Hedonism Predicts 12-Month Weight Loss After Roux-en-Y Gastric Bypass

**DOI:** 10.1007/s11695-017-2603-z

**Published:** 2017-02-22

**Authors:** Sven Alfonsson, Sandra Weineland-Strandskov, Magnus Sundbom

**Affiliations:** 1 Department of Women’s and Children’s Health, Uppsala University, Box 572, 751 23 Uppsala, Sweden; 2 Centre for Psychiatry Research, Department of Clinical Neuroscience Karolinska Institutet & Stockholm Health Care Services, Stockholm, Sweden; 3 Närhälsan, Research and Development Center, Primary Health Care, Södra Älsvborg, Borås, Sweden; 4 Department of Surgical Sciences, Uppsala University, Uppsala, Sweden

**Keywords:** Bariatric surgery, Weight loss, Risk factors, Time perspective, Personality, Hedonism

## Abstract

**Introduction:**

Research regarding psychological risk factors for reduced weight loss after bariatric surgery has yielded mixed results, especially for variables measured prior to surgery. More profound personality factors have shown better promise and one such factor that may be relevant in this context is time perspective, i.e., the tendency to focus on present or future consequences. The aim of this study was to investigate the predictive value of time perspective for 12-month weight loss after Roux-en-Y gastric bypass surgery.

**Methods:**

A total of 158 patients were included and completed self-report instruments prior to surgery. Weight loss was measured after 12 months by medical staff. Background variables as well as self-reported disordered eating, psychological distress, and time perspective were analyzed with regression analysis to identify significant predictors for 12-month weight loss.

**Results:**

The mean BMI loss at 12 months was 14 units, from 45 to 30 kg/m^2^. Age, sex, and time perspective could significantly predict weight loss but only male sex and self-reported hedonism were independent risk factors for reduced weight loss in the final regression model.

**Conclusion:**

In this study, self-reported hedonistic time perspective proved to be a better predictor for 12-month weight loss than symptoms of disordered eating and psychological distress. It is possible that a hedonistic tendency of focusing on immediate consequences and rewards is analogous to the impaired delay discounting seen in previous studies of bariatric surgery candidates. Further studies are needed to identify whether these patients may benefit from extended care and support after surgery.

## Introduction

Despite the proven effectiveness of bariatric surgery, 5–9% of bariatric surgery patients experience an unsatisfactory long-term weight loss [1, 2]. Although bariatric surgery is the most effective treatment modality at present, weight regain leads to weakened remission of comorbid diseases such as diabetes and unnecessary costs [3]. Unfortunately, predictive variables for unsuccessful weight loss are lacking.

The search for preoperative risk factors has mainly focused on eating disorders and other axis-I diagnosis and resulted mixed results [4, 5]. Treatment adherence and cognitive function predict weight loss after surgery, and taken together, this suggests that an inability to govern behavior and perform effective self-care may be important risk factors to assess in bariatric surgery patients [6–8]. Treatment adherence is influenced by demographical variables such as gender, age, and education [9]. It has been hypothesized that two factors play a crucial role: sensation seeking and reduced inhibitory control [10, 11]. Sensation seeking is largely driven by dopamine and serotonergic systems that promote exploratory behavior in new environments while inhibitory control are prefrontal processes that can stop or postpone inappropriate impulsive or pleasure seeking behavior. It has been suggested that reduced executive function may be a risk factor for bariatric surgery patients but this needs further research [7, 12].

A few studies have investigated more profound psychological variables, such as Axis II personality disorders, but the mechanisms through which such disorders would affect weight loss and self-care after surgery are unclear [13, 14]. However, a personality factor that may be highly relevant in this context is time perspective, i.e., a person’s habitual tendency to focus on either past, present, or future events and consequences [15, 16]. In the famous marshmallow experiment, children were presented with a marshmallow and instructed that they were allowed to eat it immediately, but if they waited some time they would get an additional marshmallow to eat. The children who managed the temptation and postpone the reward were later found to achieve higher levels of education and to have better health in adulthood. After this study, Zimbardo and others have suggested a model in which people are characterized by having either a future-oriented, hedonistic, or fatalistic time perspective [17]. A person with a future-oriented time perspective is goal-driven, shows a pattern of postponing reward, and report high levels of health behaviors while the opposite is true for people with a hedonistic time perspective who are highly influenced by immediate consequences and events [16, 18]. Lastly, a person with a fatalistic time perspective largely ignores consequences and shows more irresponsible behaviors and health-destructive habits [19, 20]. The construct of time perspective is possible to measure by self-report and could potentially be a stable personality trait and risk factor in bariatric surgery patients [17].

The aim of this study was to assess the predictive value of time perspective for weight loss after bariatric surgery. It was hypothesized that a future-oriented time perspective measured prior to surgery would be a positive predictor of weight loss after surgery while a hedonistic or fatalistic time perspective would be negative predictors for weight loss 12 months after surgery, after controlling for disordered eating and psychological distress.

## Methods

### Procedure and Participants

Bariatric surgery patients were approached at their first visit to a major surgery clinic and informed about the study. Those who choose to participate and were eligible for Roux-en-Y Gastric Bypass (RYGBP) surgery were asked by a nurse to fill out the instruments. Data was collected 3 months prior to surgery (mean days = 87 (SD = 28)) and at follow-up appointments 12 months later (mean days = 368 (SD = 41). Participants were instructed to follow a low-calorie diet at home 4 weeks prior to surgery as part of the clinic’s preoperative routine but were not provided with other interventions during this period. All participants were informed about the study and provided written consent. Approval for the study procedure was obtained from the Regional Ethics Committee Board.

Of the 224 patients approached, 191 (85%) chose to participate and 158 (71%) provided complete data for analyses. Background variables for participants can be seen in Table 1.

**Table 1:** Participant background variables (*n* = 158)

	Mean (SD)
Age (years)	47.5 (9.02)
Sex**,** women	101 (64%)
Marital status	
Married/cohabitant	98 (62%)
Single	46 (29%)
Other	14 (9%)
Education	
Elementary/middle-school, 9 years	36 (23%)
High-school/College, 9–12 years	76 (48%)
College/university, > 12 years	46 (30%)
Occupation	
Employed	99 (63%)
Unemployed	13 (8%)
On sick-leave	35 (22%)
Retired	9 (6%)
Studying	2 (1%)

Values are presented in *N*(%) unless stated otherwise

The 33 participants who did not provide complete data and were removed from the analyses typically did not attend the follow-up appointment and were significantly more often men (*p* = .02) and had a lower level of education (*p* = .04) than participants with complete data.

### Measures

Disordered eating was measured with the General Food Cravings Questionnaire Trait (GFCQT; [21]), which provides a total score and four subscales: loss of control, emotional cravings, positive expectations, and occupation with food. The longer version of the instrument, the FCQT, has shown adequate psychometric properties in the bariatric surgery population [22]. Psychological distress was measured with the Hospital Anxiety and Depression Scale (HADS; [23]) which contains two subscales, Anxiety (HADS-A) and Depression (HADS-D) and has shown adequate psychometric properties [24]. Time perspective was measured with the Zimbardo Time Perspective Inventory [17, 25]. The original ZTPI includes 64 items and since this was deemed too cumbersome for participants in this study, the short-form version of the ZTPI with 22 items was used instead. The short-form ZTPI is scored on a five-point scale and has three subscales: Future, Hedonistic, and Fatalistic [26]. The ZTPI has been evaluated for research in health psychology and shown adequate psychometric properties [27].

Body mass index (BMI) was calculated from weight and length, which was measured at the clinic by the medical staff.

### Analysis

Before analysis, data were screened for outliers and normality, linearity, and homoscedasticity were evaluated by scrutinizing the residual scatterplots between predicted variables and errors of prediction and found to be adequate. Because subscales were entered into the analyses, multicollinearity was assessed by analyzing the variance inflation factor for each predictor variable and found to be non-problematic.

Bivariate regression analyses were first used to identify candidate (*p* < .10) predictor variables for each outcome variable. All identified predictor variables were included in subsequent multiple linear regression analyses using a backward deletion process for each outcome variable. *R* ^2^ was used as a measure of overall model fit. The sample size of 158 was deemed adequate for multivariate regression analysis of a maximum of seven predictor variables. The predictor variables consisted of the background variables age, sex (female = 0, male = 1), level of education (9 years, 9–12 years, >12 years), and the self-report instruments GFCQT, HADS, and ZTPI. Pre-surgery BMI was included as a covariate in all regression analyses. The two main outcome variables in this study were BMI loss and %BMI loss, calculated for each participant based on the BMI measured 3 months before surgery and at a 12-month follow-up.

Correlation was assessed with Pearson’s *r* and differences between groups were analyzed with *t* tests. A *p* value less than .05 was considered statistically significant in all analyses. Missing data on individual items were imputed with expected maximization simulation. Statistical software *R* was used to conduct all analyses.

## Results

The mean BMI was 44.6 (SD = 5.8, range 34.0–56.3) before surgery and 30.2 (SD = 4.4, range 21.1–40.5) at a 12-month follow-up. The mean difference in BMI between the two time points was 14.4 (SD = 4.6, range 3.4–27.1), which corresponded to a 32% (SD = 8, range 9–50) BMI loss. The mean values and standard deviation of all self-report measures prior to surgery can be seen in Table 2.

**Table 2:** Self-report measures prior to surgery (*n* = 158)

Instrument	M (SD)
GFCQT	
Preoccupation	2.05 (0.98)
Loss of control	2.38 (1.09)
Positive expectancies	2.19 (1.01)
Emotional Cravings	1.93 (1.03)
HADS	
Anxiety	3.99 (3.75)
Depression	4.20 (4.00)
ZTPI	
Future	3.07 (0.49)
Hedonistic	2.33 (0.36)
Fatalistic	2.79 (0.46)

*GFCQT* General Food Cravings Questionnaire Trait, *HADS* Hospital Anxiety and Depression Scale, *ZTPI* Zimbardo Time Perspective Inventory

In the initial bivariate regression analyses, only the background variables sex and age and the ZTPI Future and Hedonistic subscales were candidate predictor variables for BMI loss and %BMI loss, see Table 3. These four variables were entered into a multivariate regression analysis which showed that only sex and ZTPI Hedonistic subscale could significantly predict BMI loss and %BMI loss at a 12-month follow-up. In these final multivariate models, *R* ^2^ equaled .55 for BMI loss and .23 for %BMI loss.

**Table 3:** Candidate variables in the bivariate analyses and the final multivariate model (*n* = 158)

	BMI loss				%BMI loss			
	*B (SE)*	*Beta*	*t*	*p*	*B*	*Beta*	*t*	*p*
Bivariate analyses								
Age	−0.09 (0.04)	−.18	−2.39	.02	−0.01 (0.01)	−.24	−2.44	.02
Sex	−2.31 (0.76)	−.23	−3.03	.01	−0.05 (0.02)	−.29	−3.00	.01
Education	0.54 (0.54)	.09	0.99	.33	0.12 (0.01)	.12	0.99	.33
GFCQT								
Preoccupation	−0.24 (0.38)	−.05	−0.63	.53	−0.01 (0.01)	−.07	−0.71	.48
Loss of control	−0.48 (0.35)	−.11	−1.35	.18	−0.01 (0.01)	−.13	−1.33	.19
Positive expectancies	−0.54 (0.42)	−.10	−1.28	.20	0.02 (0.01)	−.16	−1.56	.12
Emotional cravings	−0.53 (0.39)	−.11	−1.37	.17	−0.01 (.01)	−.16	−1.53	.13
HADS								
Anxiety	−0.03 (0.10)	−.03	−0.32	.75	−0.01 (0.01)	−.06	−0.58	.56
Depression	−0.03 (0.09)	−.02	−0.29	.77	−0.01 (0.01)	−.04	−0.43	.67
ZTPI								
Future	1.35 (0.65)	.16	2.09	.04	0.03 (0.02)	.20	2.04	.04
Hedonistic	−2.33 (0.90)	−.20	−2.58	.01	−0.05 (0.02)	−.22	−2.26	.03
Fatalistic	1.03 (0.76)	.11	1.36	.18	0.03 (0.02)	.15	1.52	.13
Multivariate analysis								
Sex	−2.31 (0.74)	−.23	−3.15	.002	−0.05 (0.02)	−.29	−3.08	.003
ZTPI Hedonistic	−2.33 (0.84)	−.20	−2.78	.007	−0.05 (0.02)	−.23	−2.41	.018

*GFCQT* General Food Cravings Questionnaire Trait, *HADS* Hospital Anxiety and Depression Scale, *ZTPI* Zimbardo Time Perspective Inventory

In post hoc analyses, it was found that the BMI loss and %BMI loss were 12.2 and 28% for men and 15.2 and 34% for women, respectively. A similar pattern was seen for the quartile of participants that scored the highest on the ZTPI Hedonistic subscale whose BMI loss and %BMI loss were 12.7 and 29% as compared to 14.9 and 33% among other participants.

## Conclusion

The aim of this study was to assess the predictive value of time perspective for weight loss after bariatric surgery. In line with our hypotheses, the ZTPI Future and Hedonistic subscales could predict weight loss in bivariate analyses. However, in the subsequent multivariate analyses including age, sex, and the ZTPI Future and Hedonistic subscales as well as pre-surgery BMI, only sex and the ZTPI Hedonistic subscale remained as significant predictor variables for 12-month weight loss.

The main finding of this study that the ZTPI Hedonistic subscale is a better pre-surgery predictor of 12-month weight loss than symptoms of disordered eating and psychological distress is in line with previous findings in which a hedonistic time perspective is found to be associated with impulsivity and unhealthy behaviors [16, 27]. Studies have shown that adherence to follow-ups and recommendations both before and after surgery may predict weight loss up to more than 2 years after the procedure [28]. It is possible that the ZTPI Future and Hedonistic subscales in part measures variables analogue to the construct of executive function which includes planning and goal-directed behavior [29–31]. Time perspective is supposed to mirror a person’s habitual and mostly unconscious focus on either the present or future consequences of their behavior and many bariatric surgery patients probably struggle with the rather strict regimes after surgery. Self-reported hedonism may therefore be a risk factor for engaging in rewarding behaviors, such as eating energy-dense foods and avoiding behaviors with few immediate rewards, such as physical exercise [32]. A hedonistic time perspective has previously been associated with behaviors such as drinking alcohol and inadequate dietary choices which have been highlighted as risk factors after bariatric surgery [20]. However, one should be careful when interpreting this data since there is a prejudice that people with obesity are hedonistic, lack self-control, and therefore are themselves to blame for their weight. Since obesity develops under the influence of various genetic, environmental, and behavioral factors, the purpose of this study is to elucidate methods to better identify patients who are at risk of inferior weight loss so that they can be offered adequate care and support after surgery.

The results from the present study regarding symptoms of disordered eating and psychological distress are in line with previous studies showing that psychological problems prior to surgery is a poor risk indicator for weigh loss [33]. Age and sex have been shown to be associated with weight loss after surgery in some previous studies but results have been mixed [34, 35]. In the present study, men had lost less weight after 12 months than women but the reasons for sex differences among bariatric surgery candidates are unknown [36, 37].

A weakness of the present study is the reliance on self-report measurements. While the included instruments are widely used in research, it is difficult to assess the validity of the measured constructs, especially for the ZTPI. On the other hand, using computerized tests to measure the concept of time perspective objectively is not feasible in most clinical settings, so self-report instruments such as the ZTPI may have a role to play in both research and clinical care. A further limitation is that self-report data was only collected prior to surgery. Follow-up measurements would have made it possible to conduct more advanced analyses but this was unfortunately not possible in this project. However, as data was collected when participants had been accepted for surgery, the risk of patients’ down-playing symptoms of disordered eating and psychological distress, due to a misinformed fear of being rejected surgery, seems low.

In conclusion, this study showed that male sex and a self-reported hedonistic time perspective prior to surgery were independently negatively associated with weight loss at a 12-month follow-up. Self-reported hedonism was in this study a better predictor for weight loss than symptoms of disordered eating and psychological distress. A hedonistic time perspective characterized by a tendency to focus on behaviors’ immediate consequences may therefore be a risk factor to assess in pre-surgery screening in order to tailor patient care and support. Future studies may further elucidate the associations between self-reported hedonism on the one hand and variables such as impulsivity, executive function, and treatment adherence on the other.
